# Role of Sphingosine 1-Phosphate Signalling Axis in Muscle Atrophy Induced by TNFα in C2C12 Myotubes

**DOI:** 10.3390/ijms22031280

**Published:** 2021-01-28

**Authors:** Caterina Bernacchioni, Veronica Ghini, Roberta Squecco, Eglantina Idrizaj, Rachele Garella, Elisa Puliti, Francesca Cencetti, Paola Bruni, Chiara Donati

**Affiliations:** 1 Department of Experimental and Clinical Biomedical Sciences “M. Serio”, University of Florence, 50134 Florence, Italy; elisa.puliti@unifi.it (E.P.); paola.bruni@unifi.it (P.B.); chiara.donati@unifi.it (C.D.); 2 Magnetic Resonance Center (CERM), University of Florence, 50019 Sesto Fiorentino, Italy; ghini@cerm.unifi.it; 3 Consorzio Interuniversitario Risonanze Magnetiche di Metallo Proteine (CIRMMP), 50019 Sesto Fiorentino, Italy; 4 Department of Experimental and Clinical Medicine, Section of Physiological Sciences, University of Florence, 50134 Florence, Italy; roberta.squecco@unifi.it (R.S.); eglantina.idrizaj@unifi.it (E.I.); rachele.garella@unifi.it (R.G.)

**Keywords:** sphingosine 1-phosphate, skeletal muscle atrophy, tumor necrosis factor alpha, sphingosine 1-phosphate receptors, NMR metabolomics, electrophysiological properties, autophagy

## Abstract

Skeletal muscle atrophy is characterized by a decrease in muscle mass causing reduced agility, increased fatigability and higher risk of bone fractures. Inflammatory cytokines, such as tumor necrosis factor-alpha (TNFα), are strong inducers of skeletal muscle atrophy. The bioactive sphingolipid sphingosine 1-phoshate (S1P) plays an important role in skeletal muscle biology. S1P, generated by the phosphorylation of sphingosine catalyzed by sphingosine kinase (SK1/2), exerts most of its actions through its specific receptors, S1P_1–5_. Here, we provide experimental evidence that TNFα induces atrophy and autophagy in skeletal muscle C2C12 myotubes, modulating the expression of specific markers and both active and passive membrane electrophysiological properties. NMR-metabolomics provided a clear picture of the deep remodelling of skeletal muscle fibre metabolism induced by TNFα challenge. The cytokine is responsible for the modulation of S1P signalling axis, upregulating mRNA levels of S1P_2_ and S1P_3_ and downregulating those of SK2. TNFα increases the phosphorylated form of SK1, readout of its activation. Interestingly, pharmacological inhibition of SK1 and specific antagonism of S1P_3_ prevented the increase in autophagy markers and the changes in the electrophysiological properties of C2C12 myotubes without affecting metabolic remodelling induced by the cytokine, highlighting the involvement of S1P signalling axis on TNFα-induced atrophy in skeletal muscle.

## 1. Introduction

Skeletal muscle atrophy is characterized by a decrease in muscle mass and fiber size as a result of different conditions such as aging, bed rest, cancer, denervation and motor neuron disease [1]. Skeletal muscle atrophy causes reduced agility, increased fatigability and higher risk of bone fractures, thus representing a major burden for health systems, lowering response to treatments and decreasing life expectancy. Although the knowledge on muscle wasting has been advanced during the last 10 years, the dissection of the molecular signalling pathways regulating skeletal muscle atrophy will pave the way for innovative interventions for the discovery of drugs to prevent this pathological condition, which are crucially relevant for neuromuscular diseases, muscle disuse and aging. The regulation of muscle mass depends on the balance between protein synthesis and degradation: during muscle atrophy, the main degradation pathways of the cell are activated. Inflammation potently induces muscle wasting and cachexia. Inflammatory cytokines, tumor necrosis factor (TNF) α in particular, are strong inducers of skeletal muscle atrophy. Increased efflux of nitrogen and amino acid from skeletal muscle and loss of body protein have been shown in mice administered with TNFα [2,3,4,5] and in pathological conditions characterized by elevated endogenous TNFα like cancer [6] or experimental sepsis [7]. Li et al. have shown that TNFα is capable of decreasing protein content and activating ATP-dependent proteolysis [8]. The cytokine promotes protein degradation mainly through ROS formation and NFkB activation [9]. Moreover, TNFα plays a crucial role in the metabolic dysregulation related to altered lipid and carbohydrate metabolism that takes place in atrophic conditions driving to increase systemic stress and energy expenditure [10,11].

TNFα, by binding to its specific receptors, activates multiple downstream signalling pathways [12] including the hydrolysis of membrane sphingomyelin, leading to the formation of bioactive sphingolipids such as ceramide and sphingosine [13,14]. The ATP-dependent phosphorylation of sphingosine catalyzed by two different isoforms of sphingosine kinase (SK), SK1 and SK2, then generates sphingosine 1-phosphate (S1P). S1P is a pleiotropic molecule that is crucially involved in the regulation of both physiological and pathological processes [15,16]. The catabolism of S1P is finely regulated since S1P can be irreversibly cleaved by S1P lyase (SPL) to hexadecenal and phosphoethanolamine or can be reverted back to sphingosine by the action of specific S1P phosphatases (SPP) and non-specific lipid phosphate phosphatases (LPP). The mechanism of action of S1P is dual since it can act as an intracellular messenger and as a ligand of five different specific G protein-coupled receptors (S1PR), named S1P_1-5_ [17]. The release in the extracellular environment of S1P is mediated through the specific transporters spinster homolog 2 (Spns2) [18] and Mfsd2b [19] or unspecific transporters belonging to the ATP-binding cassette (ABC) family [20]. S1P signalling plays an important biological role in skeletal muscle [21]. S1P has been shown to be involved in satellite cell activation [22,23] and to improve muscle regeneration in injured *mdx* mice [24]. In C2C12 myoblasts, S1P acts as negative regulator of cell proliferation and migration and as a powerful activator of myogenic differentiation [25,26]. Moreover, the SK/S1P axis in myoblasts appears to be under the control of multiple extracellular cues that exploit this signalling pathway to elicit specific biological responses such as IGF-1 and PDGF [27,28]. The sphingolipid significantly reduced the tension decline during fatigue of skeletal muscle [29] and exerted a trophic action in denervated rat soleus muscle [30]. We previously showed that low doses of TNFα promote myogenesis in C2C12 myoblasts and inhibition of SK and S1P_2_ abrogated its pro-myogenic effect [31]. TNFα at high doses induces muscle wasting, essentially by interfering with the ability of satellite cells to differentiate into myofibres [32]. The biosynthesis of ceramide induced by TNFα has been shown to be involved in the mechanisms leading to muscle loss associated with pathological states [33]. Here, we provide experimental evidence that TNFα dose-dependently induces atrophy and autophagy in skeletal muscle C2C12 myotubes, downregulating the protein level of myosin heavy chain (MHC), upregulating the autophagy marker LC3-II and modulating both active and passive electrophysiological membrane properties. Untargeted ^1^H-NMR metabolomics provided a clear picture of the deep remodelling of skeletal muscle fibre metabolism induced by TNFα challenge. S1P signalling and metabolism were modulated by the cytokine, being responsible for the upregulation of S1P_2_/S1P_3_ mRNA levels and the downregulation of SK2 transcript. Short time of incubation with the cytokine increases the phosphorylated form of SK1, readout of its activation. Interestingly, pharmacological inhibition of SK1 and specific antagonism of S1P_3_ prevented the increase in LC3-II and the changes in the electrophysiological properties of C2C12 myotubes induced by the cytokine, without affecting the metabolic remodelling, thus highlighting the involvement of S1P signalling axis in TNFα-induced atrophy in skeletal muscle.

## 2. Results

**Figure 1:**
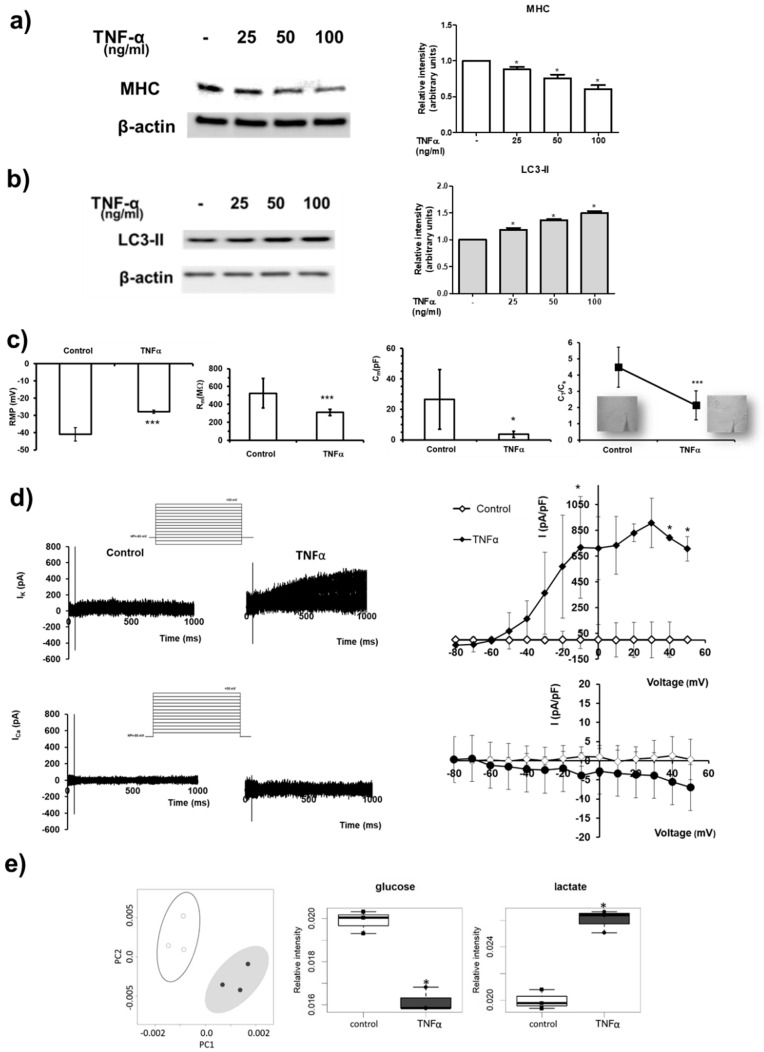
TNFα-induced atrophy in C2C12 myotubes. (**a**,**b**) C2C12 myotubes were treated with TNFα at the indicated concentrations (25–100 ng/mL) for 24 h. Western blot analysis was performed using specific anti-MHC (**a**) and anti-LC3 (**b**) antibodies in cell lysates. Left panels: blot representative of at least three independent experiments with analogous results is shown. Right panels: densitometric analysis of at least three independent experiments. Data are the mean ± SEM and are reported as protein expression normalized to β-actin, -fold change over control (set as 1). TNFα decreases the expression of MHC in a statistically significant manner by one-way ANOVA followed by Bonferroni’s post hoc test (* *p* < 0.05, treated vs. control). TNFα increases LC3-II in a statistically significant manner by one-way ANOVA followed by Bonferroni’s post hoc test (* *p* < 0.05, treated vs. control). (**c**) Resting membrane potential (RMP) evaluated in current-clamp mode, membrane resistance (R_m_) and membrane capacitance (C_m_ ) estimated in voltage-clamp and C_T_/C_s_ ratio in the different conditions. All of the results and number of cells investigated are listed in Table S1 as mean ± SD. * indicates *p* < 0.05 and *** *p* < 0.001 vs. control (unpaired Student’s *t* test). (**d**) Upper panels: representative outward K^+^ currents (in pA) evoked in response to 1-s long pulse protocol ranging from −80 up to +50 mV (HP = −60 mV) in control myotubes and in TNFα-treated ones. Overall I–V plots related to all the experiments conducted in any conditions are reported. The current amplitude is measured for each voltage step at the end of the pulse. * *p* < 0.05 vs. control (unpaired Student’s *t* test), (*n* = 6 to 7 for the different treatments). Lower panels: typical inward Ca^2+^ currents (in pA) recorded using the solutions described in the Methods section in response to 1-s long pulse protocol from −80 up to +50 mV (HP = −80 mV) in control myotubes and in TNFα-treated ones. Overall I–V plots related to the amount of experiments conducted in any conditions show not statistically significant differences (*p* > 0.05 vs. control, unpaired Student’s *t* test), (*n* = 4 to 7, for the different treatments). Data are the mean ± SD. Error bars are shown when their size is bigger than the symbol. (**e**) Left panel: metabolomic phenotyping of conditioned media (exo-metabolome) of C2C12 myotubes treated or not with 100 ng/mL TNFα for 24 h. Score plots of PCA: PC1 vs. PC2. In the score plots, each dot represents a different sample, and each colour represents a different group of samples—white: control; dark grey: TNFα. Right panels: boxplot of the metabolites involved in glycolysis. Changes in metabolite levels caused by TNFα were statistically significant by paired Wilcoxon test, (* *p* < 0.05).

The atrophic effect of TNFα was analysed in C2C12 myotubes. Treatment with increasing concentrations of the cytokine (25, 50, 100 ng/mL) for 24 h induced muscle protein degradation, as demonstrated by the dose-dependent reduction in the structural protein MHC (Figure 1a). Moreover, TNFα induced autophagic proteolysis since the treatment with the cytokine caused a dose-dependent increase in the lipidated form of LC3, LC3-II, accepted as the gold standard for determining autophagosome formation (Figure 1b).

To evaluate the action of TNFα on myotube functional aspects, membrane phenomena related to the excitability and excitation-contraction coupling were analysed (Figure 1c, Table S1). TNFα treatment caused a statistically significant depolarization of the membrane (i.e., increase in RMP, resting membrane potential) compared to control myotubes. To test the possible effect of TNFα on membrane permeability, membrane resistance, R_m_, was measured. The R_m_ value significantly decreased in TNFα-treated myotubes, suggesting a leakier membrane under atrophic challenge. The analysis of cell capacitance (C_m_), assumed as index of myotube surface membrane, showed a clear significant decrease in the presence of TNFα compared to control myotubes. Accordingly, to assess if the observed C_m_ decrease was due to a reduction in the T-tubular system, we used a previously published procedure [34]. The ratio between the capacitance associated with the tubular membrane C_T_ and the capacitance associated with the surface membrane C_s_, C_T_/C_s_, was significantly smaller under TNFα treatment compared to control. Moreover, the effect of TNFα on myotube ion currents (Figure 1d) crucial in the control of the excitability and Ca^2+^ entry in muscle cells was tested. The K^+^ currents evoked in myotubes normally showed a small amplitude. TNFα caused a large and statistically significant increase in outward current size. Representative traces are depicted in Figure 1d for control and TNFα treatment, respectively. Similarly, Ca^2+^ currents showed a very small amplitude. However, TNFα did not significantly alter the inward current size.

The metabolic shift induced by TNFα in skeletal muscle was characterized by comparing the extracellular metabolomic profiles (exo-metabolome) of TNFα-treated vs. control myotubes. Exo-metabolome provided steady-state levels of metabolites resulting from the balance between production and consumption of each molecule; unsupervised principal component analysis (PCA) was used to obtain a preliminary overview of the data (Figure 1e). From the PCA score plot (PC1 vs. PC2), a discrimination between the two different conditions tested (TNFα-treated vs. control myotubes) was clearly visible, indicating a strong influence of the cytokine on myotube metabolic profiles. In particular, treatment with 100 ng/mL TNFα for 24 h induced a metabolic shift towards aerobic glycolysis, characterized by significantly reduced extracellular glucose levels and increased levels of lactate (Figure 1e). In addition, a marked reduction in the extracellular aminoacid content was observed (Figure S1).

**Figure 2:**
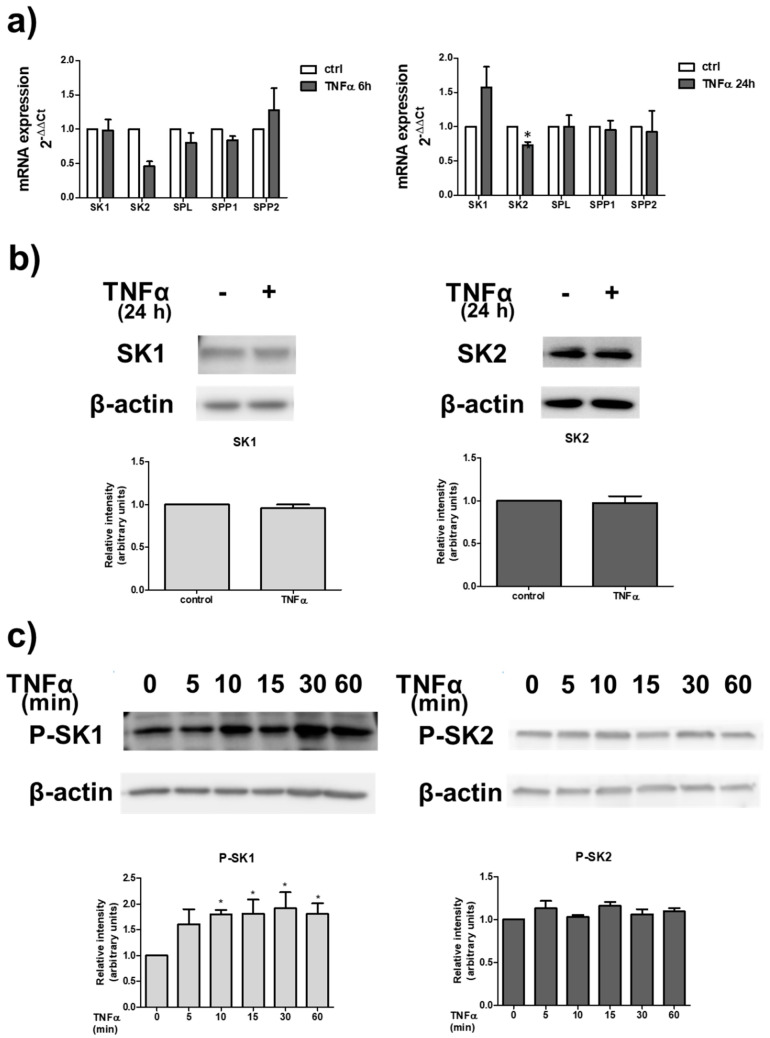
TNFα modulates S1P metabolism. (**a**) Quantitative mRNA analysis was performed by real-time PCR in total RNA extracted from C2C12 myotubes stimulated or not with 100 ng/mL TNFα for the indicated time intervals (6 h and 24 h). mRNA quantitation of S1P metabolism enzymes (SK1, SK2, SPL, SPP1 and SPP2) was based on the 2^−ΔΔCt^ method, using individual enzyme of the unchallenged specimen as calibrator. Data are the mean ± SEM of three independent experiments performed in triplicate. The reduction in SK2 expression induced by TNFα was statistically significant by Student’s *t* test (* *p* < 0.05). (**b**,**c**) C2C12 myotubes were incubated for the indicated time intervals in the absence or in the presence of 100 ng/mL TNFα. (**b**) Top, aliquots of total cell lysates were used to perform Western analysis, using specific anti-SK1 and anti-SK2 antibodies. A representative blot is shown. Bottom, densitometric analysis of at least three independent experiments. Data are the mean ± SEM and are reported as protein expression normalized to β-actin, -fold change over control (set as 1). (**c**) Western blot analysis was performed using specific anti-phospho-SK1 and anti-phospho-SK2 antibodies. A blot representative of at least three independent experiments with analogous results is shown. The histogram represents densitometric analysis of three independent experiments. Data are the mean ± SEM and are reported as protein expression normalized to β-actin, -fold change over control (set as 1). The increase in phospho-SK1 content induced by TNFα was found to be statistically significant by one-way ANOVA followed by Bonferroni’s post hoc test (* *p* < 0.05, treated vs. control).

The possible involvement of S1P signalling axis in the atrophic effect of TNFα in skeletal muscle was investigated. To this aim, we first examined whether the cytokine was capable of regulating the expression of enzymes implicated in S1P metabolism in C2C12 myotubes. Real Time PCR data illustrated in Figure 2a show that the treatment with 100 ng/mL TNFα significantly reduced the mRNA expression of SK2 at 24 h of treatment while the expression of other enzymes involved in S1P metabolism was unaffected by the treatment with the cytokine for 6 or 24 h. We then investigated whether TNFα was able to regulate the protein content of SK1 and SK2. Western blot analysis of SKs levels showed that 100 ng/mL TNFα for 24 h did not modulate SK1 or SK2 protein levels in myotubes (Figure 2b). Since agonist-induced stimulation of SK1/SK2 activity and translocation to the plasma membrane is mediated by phosphorylation [35,36], Western blot analysis using specific anti-phospho-SK1 as well as anti-phospho-SK2 antibodies was performed in myotube lysates following TNFα treatment. Data reported in Figure 2c showed that cell challenge with 100 ng/mL TNFα provoked a rapid increase in SK1 phosphorylation, detectable at 10 min of incubation and persistent after 60 min of treatment. In contrast, the phosphorylation state of SK2 was unaffected by TNFα treatment. Collectively, these data indicate that although TNFα does not alter SK protein expression, it is able to rapidly and persistently activate SK1 in myotubes.

**Figure 3:**
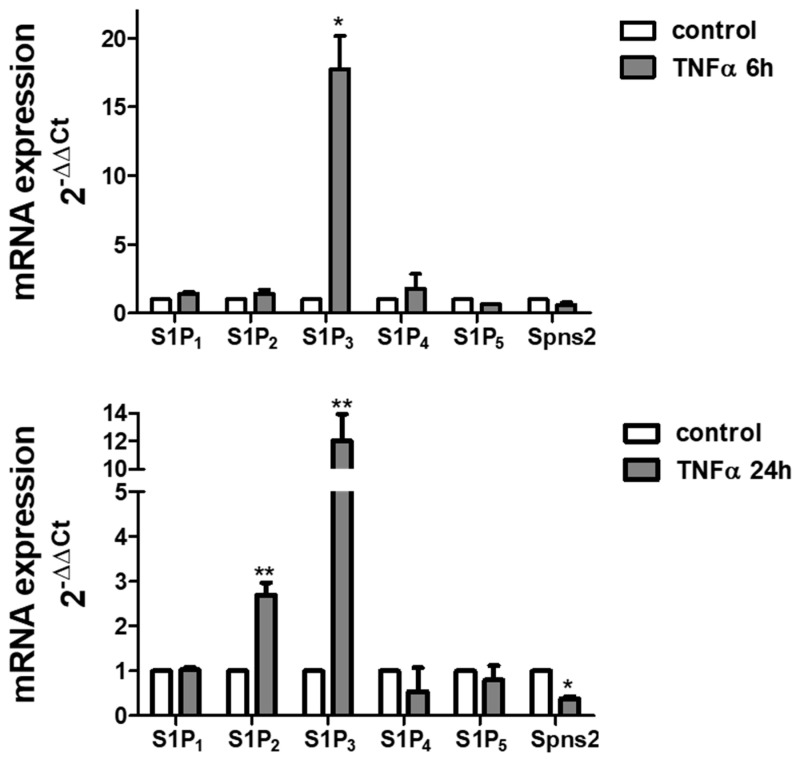
TNFα modulates S1P signalling. Quantitative mRNA analysis was performed by real-time PCR in total RNA extracted from C2C12 myotubes stimulated or not with 100 ng/mL TNFα for the indicated time intervals (6 h and 24 h). mRNA quantitation of S1PR (S1P_1_, S1P_2_, S1P_3_, S1P_4_ and S1P_5_) and S1P specific transporter, Spns2, was based on the 2^−ΔΔCt^ method, using individual enzyme or Spns2 of the unchallenged specimen as calibrator. Data are the mean ± SEM of three independent experiments performed in triplicate. The increase in S1P_2_ and S1P_3_ and the decrease in Spns2 expression induced by TNFα were statistically significant by Student’s *t* test (* *p* < 0.05; ** *p* < 0.01).

The effect of TNFα treatment on S1P signalling was analysed to determine the effect of the cytokine on S1PR expression. The treatment with TNFα deeply modulates S1PR expression pattern in myotubes: quantitative analysis of mRNA demonstrated that the cytokine potently augmented S1P_2_ levels (about 2.5-fold increase at 24 h of treatment) and S1P_3_ levels (more than 10-fold increase both at 6 h and 24 h of treatment) (Figure 3). In addition, the cytokine significantly reduced the mRNA levels of Spns2 after 24 h challenge.

**Figure 4:**
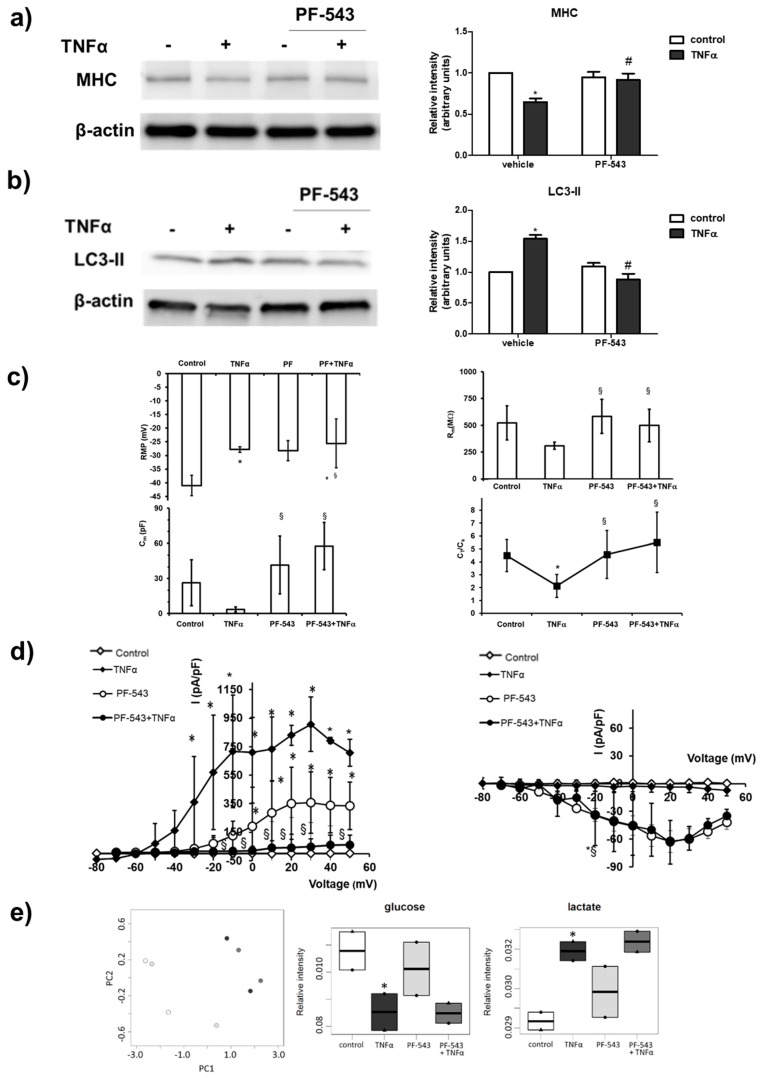
SK1 is involved in TNFα-induced effect in C2C12 myotubes. C2C12 myotubes were pre-treated with SK1 specific inhibitor PF-543 (10 µM) for 1 h before being challenged with 100 ng/mL TNFα for 24 h. The content of MHC (**a**) and LC3-II (**b**) was analyzed by Western blotting of whole cell lysates. A blot representative of three independent experiments with analogous results is shown. The histograms represent the densitometric analysis of at least three independent experiments; Data are the mean ± SEM and are reported as protein expression normalized to β-actin, -fold change over control (set as 1). The effect of SK1 inhibition by PF-543 on TNFα atrophic effect is statistically significant by two-way ANOVA followed by Bonferroni’s post hoc test (# *p* > 0.05). (**c**) RMP, R_m_, C_m_ and C_T_/C_s_ ratio in the different conditions. All of the results and number of cells investigated are listed in Table S1 as mean ± SD. * Indicates *p* < 0.05 vs. control; § indicates *p* < 0.05 vs. TNFα; (One-way ANOVA and Bonferroni’s post hoc test). (**d**) Left panel: overall I–V plots related to K^+^ currents measured in all of the experiments conducted in any conditions. * *p* < 0.05 vs. control, § *p* < 0.05 PF-543 + TNFα vs. TNFα (two-way ANOVA and Bonferroni’s post hoc test) (*n* = 5 to 7 for the different treatments). Right panel: Overall I–V plots related to Ca^2+^ currents measured in all of the experiments conducted in any conditions (*n* = 4 to 7 for the different treatments). * *p* < 0.05 vs. control (two-way ANOVA and Bonferroni’s post hoc test). Error bars are shown when their size is bigger than the symbol. (**e**) Left panel: Metabolomic phenotyping of conditioned media (exo-metabolome) of C2C12 myotubes treated or not with 100 ng/mL TNFα for 24 h. Score plots of PCA: PC1 vs. PC2. In the score plots, each dot represents a different sample, and each color represents a different group of samples—white: control; dark grey: TNFα; light grey:PF-543; medium gray: PF-543 + TNFα. Right panels: boxplot of the metabolites involved in glycolysis. Changes in metabolite levels caused by TNFα were statistically significant by paired Wilcoxon test, (* *p* < 0.05).

The potential role of SK1 activation on the atrophic action induced by the cytokine was then examined. To this aim, C2C12 myotubes were treated with 10 µM PF-543, specific inhibitor of SK1. Interestingly, treatment with the inhibitor completely reverted the reduction in MHC levels induced by TNFα, suggesting a key role of SK1 in the atrophic effect of the cytokine (Figure 4a). In accordance, SK1 inhibition abolished the increase in the autophagic marker LC3-II induced by TNFα (Figure 4b). Accordingly, when TNFα was added in the presence of SK1 inhibitor PF-543, RMP was not significantly modified compared to that recorded from cells treated with TNFα alone, suggesting that this effect is barely associated with S1P formation (Figure 4c, Table S1). However, the pre-treatment with PF-543 completely reverted the effect of TNFα on R_m_, whose values became even larger than those measured in control myotubes, suggesting the involvement of SK1/S1P pathway in this TNFα-induced effect. Similarly, pre-treatment with PF-543 completely hindered the effect of TNFα on C_m_. In accordance, the C_T_/C_s_ ratio calculated from myotubes treated with TNFα when SK1 was inhibited was similar to that measured in control and statistically different to that measured with TNFα alone (Figure 1c). These results indicate that TNFα reduces the Cm, mainly affecting T-tubular surface via S1P axis. In addition, the effect of TNFα on K^+^ currents was completely reverted by SK1 inhibition, suggesting the involvement of the enzyme in this effect. These data are summarized in the I–V plots (Figure 4d) where each point represents the mean ± SD of the current values measured at the end of the test pulse for all the experiments conducted, reported as a function of the voltage step applied. Of note, the inhibition of SK1 seems to be very effective in hindering TNFα action on K^+^ currents, since data points related to this treatment (filled circles) mostly overlap the control data (open diamonds). Although TNFα does not significantly affect *I*_Ca_ occurrence in our preparations (filled diamonds), when SK1 was inhibited an increase in current amplitude, both in the absence and presence of the cytokine (Figure 4d, open and filled circles, respectively), was observed. These results demonstrate that the TNFα-mediated atrophic effect strictly depends on the activation of SK1 in skeletal muscle.

However, the obtained findings showed that SK1 is not implicated in the metabolic shift induced by TNFα. Indeed, from the PCA score plot reported in Figure 4e, it is clearly evident that the exo-metabolome fingerprint of the cells is not significantly influenced by pre-treatment with 10 µM PF-543, indicating that the blockade of SK1 does not significantly affect the glycolytic shift (Figure 4e) nor the extracellular aminoacid content (Figure S2) induced by the cytokine.

**Figure 5:**
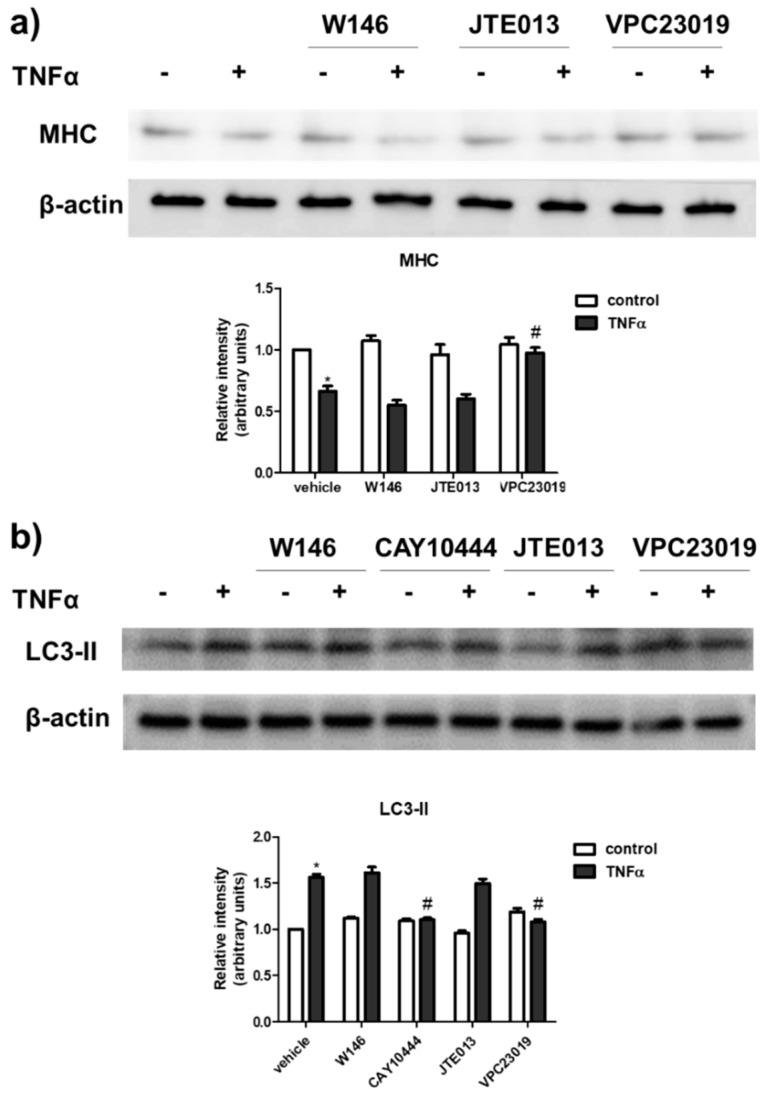
S1P_3_ is involved in TNFα-induced effect in C2C12 myotubes. C2C12 myotubes were pre-treated with S1PR specific antagonists (10 μM W146, selective antagonist of S1P_1_, or 1 μM JTE013, selective S1P_2_ antagonist, or 5 μM CAY10444, selective S1P_3_ antagonist or 1 μM VPC23019, selective S1P_1_/S1P_3_ antagonist) for 1 h before being challenged with 100 ng/mL TNFα for 24 h. The content of MHC (**a**) and LC3-II (**b**) was analysed by Western blotting of whole cell lysates. A blot representative of three independent experiments with analogous results is shown. The histograms represent the densitometric analysis of at least three independent experiments; data are the mean ± SEM and are reported as protein expression normalized to β-actin, -fold change over control (set as 1). The effect of S1P_3_ inhibition by CAY10444 or VPC23019 on TNFα atrophic effect is statistically significant by two-way ANOVA followed by Bonferroni’s post hoc test (# *p* > 0.05).

Since TNFα is responsible for a deep modulation of S1PR expression in C2C12 myotubes, we then examined whether the atrophic action exerted by the cytokine was S1PR-mediated. For this purpose, the content of MHC and LC3-II was evaluated in myotubes challenged with TNFα in the presence or absence of 10 μM W146, selective antagonist of S1P_1_, 1 μM JTE013, that selectively blocks S1P_2_, 5 μM CAY10444, selective S1P_3_ antagonist or 1 μM VPC23019, selective S1P_1_/S1P_3_ antagonist. The results illustrated in Figure 5 show that the atrophic effect of TNFα was abrogated by S1P_3_ blockade and not affected by S1P_1_ or S1P_2_ inhibition, suggesting a role of S1P_3_ in TNFα-induced response.

**Figure 6:**
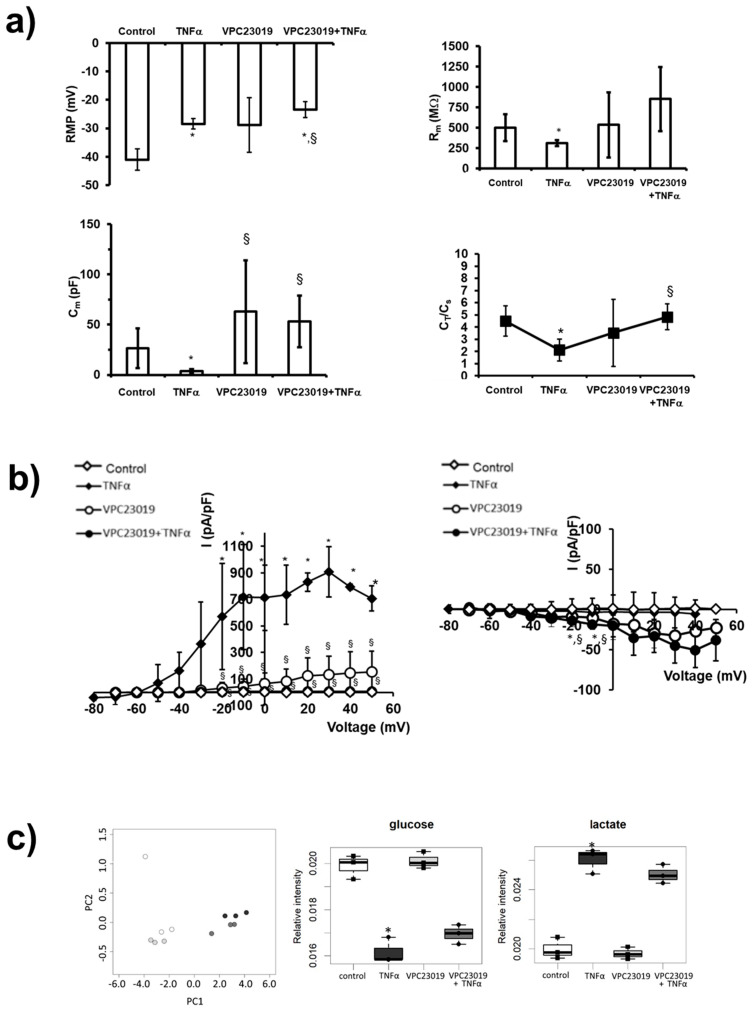
Role of S1P_3_ in TNFα-induced effect in C2C12 myotubes. (**a**) RMP, R_m_, C_m_ and C_T_/C_s_ ratio in the different conditions. All of the results and number of cells investigated are listed in Table S1 as mean ± SD. * Indicates *p* < 0.05 vs. control; § indicates *p* < 0.05 vs. TNFα; (One-way ANOVA and Bonferroni’s post hoc test). (**b**) Left panel: overall I-V plots related to K^+^ currents measured in all the experiments conducted in any conditions. * *p* < 0.05 vs. control, § *p* < 0.05 VPC23019 + TNFα vs. TNFα (two-way ANOVA and Bonferroni’s post hoc test) (*n* = 3 to 7 for the different treatments). Right panel: Overall I–V plots related to Ca^2+^ currents measured in all the experiments conducted in any conditions (*n* = 3 to 7 for the different treatments). * *p* < 0.05 vs. control; § *p* < 0.05 vs. TNFα (two-way ANOVA and Bonferroni’s post hoc test). Error bars are shown when their size is bigger than the symbol. (**c**) Left panel: Metabolomic phenotyping of conditioned media (exo-metabolome). Score plots of PCA: PC1 vs. PC2. In the score plots, each dot represents a different sample, and each colour represents a different group of samples—white: control; dark grey: TNFα; light grey: VPC23019; medium grey: VPC23019+TNFα. Right panels: boxplot of the metabolites involved in glycolysis. Changes in metabolite levels caused by TNFα were statistically significant by paired Wilcoxon test, (* *p* < 0.05).

The possible involvement of S1P_3_ in the electrophysiological changes induced by TNFα was then investigated. Figure 6a shows results obtained when TNFα was added in the presence of 1 μM VPC23019: the RMP value was not affected by the presence of the S1P_3_ antagonist since TNFα was still able to depolarize the membrane. In contrast, R_m_ values evaluated in C2C12 myotubes treated with TNFα in the presence of VPC23019 were similar to those evaluated in control myoblasts, indicating that TNFα was no more effective in altering membrane resistance when S1P_3_ was blocked. Similarly, pre-treatment with VPC23019 entirely abolished the effect of TNFα on C_m_. Accordingly, in myotubes where S1P_3_ was blocked, the treatment with TNFα did not determine a significantly different C_T_/C_s_ ratio in respect to control but was significantly higher compared to that estimated from C2C12 myotubes treated with TNFα alone. These results show that TNFα distresses the T-tubular surface via S1P_3_ signalling.

Moreover, TNFα effect on the outward currents was hindered by VPC23019 pre-treatment (Figure 6b): the data points related to the current amplitudes recorded from TNFα-treated myotubes in the presence of VPC23019 (filled circles) are not distinct from those obtained in control (open diamonds) (Figure 6b, left I–V plot). Regarding *I*_Ca_ occurrence, cell treatment with S1P_3_ antagonist alone (open circles) caused an increase in current amplitude that was not significantly modified when TNFα was added (filled circles) (Figure 6b, right I–V plot).

The PCA score plot reported in Figure 6c shows that when S1P_3_ signalling was blocked by VPC23019 (1 μM), exo-metabolome fingerprint alteration induced by TNFα was not significantly altered. In agreement, the metabolic shift (Figure 6c) or the modulation of the extracellular aminoacid content (Figure S3) induced by TNFα were unaltered by S1P_3_ blockade.

Altogether, these data support the view that TNFα exploits the SK1-dependent inside-out S1P signalling to transactivate S1P_3_ in an autocrine/paracrine fashion in order to mediate skeletal muscle atrophy and the related functional features, but not for transducing the metabolic shift induced by the cytokine.

## 3. Discussion

Skeletal muscle represents the most abundant tissue of the human body, whose function is necessary for many fundamental biological processes from movement to respiration. A tight balance between protein synthesis and degradation is required to maintain muscle homeostasis [37]. Three main pathways are involved in skeletal muscle protein degradation: ubiquitin-mediated proteasome degradation, autophagy and calcium-activated protease calpains [37]. Literature findings support a growing interest in autophagy as a mediator of skeletal muscle atrophy [38]; recently, it has been showed that autophagy, although involved in cachexia, is not required in muscle atrophy and it appears to play a critical role in myofiber maintenance [39,40] for skeletal muscle development and regeneration [41].

Consolidated literature data support a crucial role of the bioactive sphingolipid S1P in skeletal muscle biology [21]. S1P indeed plays a crucial role in satellite cell activation [22] and proliferation [42], but also on myoblast differentiation [25] and migration [26].

In the present manuscript, we provide experimental evidence that S1P signalling is deeply modulated by TNFα, which induces atrophy in C2C12 myotubes: the cytokine was capable of up-regulating the mRNA levels of S1P_2_ and even more those of S1P_3_ at 6 h and 24 h of treatment while down-regulated SK2 transcripts at 24 h. Short time of incubation with the cytokine increased the phosphorylated form of SK1, readout of its activation. Notably, the involvement of S1P signalling axis in TNFα-induced skeletal muscle atrophy was highlighted since, when SK1 and S1P_3_ were inhibited, the autophagy marker increase and the electrophysiological changes induced by the cytokine in C2C12 myotubes were significantly altered.

TNFα, initially named cachectin, is one of the most characterized cytokines that is able to promote anorexia [43] and skeletal muscle wasting [44], mainly through the NF-kB pathway. TNFα, at high doses, has been reported to induce muscle wasting, altering the ability of satellite cells to differentiate into myotubes [32]. In this work, TNFα, at high doses, down-regulated the protein level of MHC, upregulated the autophagy marker LC3-II and was also able to induce extensive changes in the electrophysiological properties of C2C12 myotubes. TNFα challenge decreased the membrane potential RMP, capacitance C_m_ and Ca^2+^ currents. This feature is well related to muscle atrophy and is usually due to a major reduction in transverse (T) tubular area [34]. During muscle wasting, the shortage of energy metabolism may contribute to dysfunction and impairment of ion channels and Ca^2+^, which is also supported by TEM analysis showing mitochondrial decrease and misplacement [34]. Membrane potentials recorded in experiments performed in denervated fibers supported a leaky sarcolemma and an increase in intracellular Ca^2+^ concentration, thus reducing membrane excitability [45]. A reduced resting membrane resistance Rm together with the reduced muscle fibre diameter could explain the reduced excitability found in muscle of cachectic patients [46]. A recent report showed that skeletal muscle oxidation of the calcium channel RyR1 (ryanodine receptor and calcium release channel) leads to leaky channels and drives to inefficient muscle activity [47].

In the present manuscript, NMR-based metabolomics provided a clear picture of the deep remodelling of C2C12 myotubes metabolism towards aerobic glycolysis induced by TNFα challenge characterized by significantly reduced extracellular glucose content and increased levels of lactate. In addition, a marked reduction in the extracellular aminoacid content was observed. These findings are in agreement with previously reported role of TNFα in the utilization of glucose, formation of lactate [48] and nitrogen metabolism [49]. Notably, in the serum of elderly people affected by age-associated skeletal muscle atrophy, low levels of tryptophan and increased proline concentrations have been reported [50,51]. TNFα plays a crucial role in the metabolic alteration that occurs in cancer cachexia [52], causing an increase in expenditure of energy, altered lipid and carbohydrate metabolism and insulin resistance. In vitro experiments performed in skeletal muscle showed that TNFα promotes a futile cycle linked to the co-activation of phosphofructokinase-1 and fructose-1,6-bisphosphatase, resulting in ATP consumption, further promoting resting energy expenditure [48].

TNFα, at low doses, has, on the contrary, been showed to be physiologically involved in skeletal muscle adaptation to exercise and in satellite cell differentiation [53,54]. We previously demonstrated that TNFα, at low doses, enhances differentiation of murine C2C12 myoblasts, increasing the expression of the early myogenic marker myogenin and MHC at early time points of differentiation [31]. Furthermore, SK has been previously shown to mediate multiple actions of TNFα such as protection from apoptotic cell death [55], proliferation [56] and inflammation [57].

In the present study, when SK1 and S1P_3_ were pharmacologically blocked, the ability of TNFα in increasing LC3-II and reducing MHC expression levels, membrane potential, capacitance and K^+^ currents was inhibited, highlighting the involvement of S1P signalling in TNFα-induced contractile protein degradation and T-tubular surface distress. Therefore, it has been demonstrated that in myotubes, obtained by differentiating myoblasts for 5 days, TNFα, via the SK1/S1P_3_ axis, induced an increase in LC3-II that parallels the reduction in MHC provoked by the cytokine, suggesting a role of autophagy in this type of skeletal muscle atrophy. However, future experiments are needed to finely and comprehensively dissect the contribution of TNFα and S1P signalling in the complex regulation of skeletal muscle autophagy. Moreover, it cannot be excluded that the reduction in MHC levels induced by TNFα via the SK1/S1P_3_ axis could be ascribed, at least in part, to a pro-apoptotic role of the cytokine in myotubes. Pre-incubation with the SK1 inhibitor PF-543 altered Ca^2+^ currents in the absence of TNFα, supporting the hypothesis that, in control conditions, SK1 activity and the consequent S1P formation may help to prevent an excessive Ca^2+^ entry through voltage dependent Ca^2+^ channels. Remarkably, the cytokine-induced metabolic remodelling appeared not to depend on the S1P axis, at least at these times of incubation, highlighting the complexity of the mechanism of action of TNFα in skeletal muscle atrophy, only partially mediated by the sphingolipid signalling.

The here reported role of S1P axis in TNFα-induced atrophy resembles that played by the bioactive sphingolipid in TGFβ-induced fibrosis in skeletal muscle [58]. Although in myoblasts, the SK/S1P axis, via S1P_2_ engagement, is physiologically required for the achievement of myogenic differentiation of C2C12 [28,31,59], SK1 activity was shown to be a mediator of the pro-fibrotic effect of TGFβ in myoblasts [58]. Interestingly, the different biological outcome mediated by SK1/S1P signalling axis after TGFβ treatment strictly depends on the remodelling of S1PR expression pattern exerted by the cytokine: concomitantly with SK1 up-regulation, TGFβ upregulates S1P_3_ that becomes the prevailing expressed receptor, and via the S1P inside-out signalling this receptor is responsible for the transmission of the cytokine pro-fibrotic action [58].

De Larichaudy et al. previously demonstrated that ceramide mediates TNFα skeletal muscle atrophy in differentiated L6 and C2C12 myotubes [33]. They showed that in L6 myotubes, exogenous S1P exerts an antagonistic action with respect to ceramide on myotube surface and creatine kinase activity. Inhibition of SK activity by dimethylsphingosine increased the effects of TNFα, highlighting that S1P plays an opposite role of ceramide. Moreover, the authors showed that treatment with FTY720 had negative effects on myotubes [33]. S1P has been shown to act as trophic factor in skeletal muscle, being capable of delaying the progression of denervation induced atrophy [30] and improving muscle regeneration in an mdx mice model of muscular dystrophy [24]. In skeletal muscles of animals bearing the C26 tumor, a well-known model of cancer cachexia, and in C2C12 myotubes treated with dexamethasone, a down-regulation of active SK1 was observed, while an up-regulation of Spns2 and S1P_2_ occurred. Interestingly, exogenous S1P counteracted the effect of dexamethasone reducing atrogin expression, while S1P alone increased the level of the atrophy markers [60]. These contrasting literature data might depend on different experimental conditions such as time of incubations or employed concentrations. Data here reported confirm the highly versatile biological outcome of the SK1/S1P/S1PR axis in skeletal muscle cells. After TNFα treatment at high doses, a deep S1PR remodelling occurs and the cytokine exerts its atrophic effect by ligation to S1P_3_, which is profoundly upregulated by TNFα. S1PR remodelling indeed appears to be the crucial aspect to mechanistically explain the different biological outcomes brought about by TNFα. Interestingly, recent findings in prostate cancer cachectic patients showed increased levels of cytokines in circulation: serum TNFα concentration was about 6.54 pg/mL [61]. Although these levels are far from that used in the present study in our in vitro experimental setting, it should be taken into account that the local tissue levels of TNFα in skeletal muscle could considerably differ from serum concentration of cachectic patients.

The present study opens innovative therapeutic perspectives for skeletal muscle wasting management, highlighting the role of S1P signalling in TNFα induced atrophy.

## 4. Materials and Methods

### 4.1. Materials

All biochemicals, cell culture reagents, Dulbecco Modified Eagle Medium (DMEM), fetal bovine serum (FBS), protease inhibitor cocktail, bovine serum albumin (BSA) selective S1P_2_ antagonist JTE013, specific S1P_1/3_ antagonist VPC23019, specific inhibitor of SK1 PF-543, and myosin heavy chain (MHC) antibody were purchased from Merck Life Science (Burlington, MA, USA). Selective S1P_1_ antagonist W146 was from Avanti Polar Lipids (Alabaster, AL, USA). Recombinant TNFα was obtained from PeproTech (London, UK). Human specific TaqMan Gene Expression Assays employed for gene expression studies were purchased from Thermo Fisher Scientific INC (Waltham, MA, USA). Anti-SK1, anti-SK2, anti-phospho-SK1 (Ser225) and anti-phospho-SK2 (Thr578) antibodies were from ECM Biosciences (Versailles, KY, USA). Secondary antibodies conjugated to horseradish peroxidase, anti-LC3 antibody and anti-β-actin antibodies were obtained from Santa Cruz Biotechnology (Santa Cruz, CA, USA).

### 4.2. Cell Culture

Murine C2C12 myoblasts were maintained in DMEM containing 10% FCS, 2 mM l-glutamine, 100 μg/mL streptomycin and 100 U/mL penicillin at 37 °C in 5% CO_2_. To induce differentiation, confluent cells were cultured in DMEM without serum supplemented with 1 mg/mL BSA for 5 days. The myotubes obtained were then treated with 25–100 ng/mL TNFα for 24 h to induce atrophy. In some of the experiments, cells were pre-treated with 10 μM W146, 1 μM JTE013, 5 μM CAY10444, 1 μM VPC23019, 10 μM PF-543, 1 h before agonist stimulation.

### 4.3. Western Blot Analysis

C2C12 myotubes were collected and lysed for 30 min at 4 °C in a buffer containing 50 mM Tris, pH 7.5, 120 mM NaCl, 1 mM EDTA, 6 mM EGTA, 15 mM Na_4_P_2_O_7_, 20 mM NaF, 1% Nonidet and protease inhibitor cocktail before being centrifuged at 10,000× *g*, 15 min 4 °C. Samples resuspended in Laemmli’s SDS (sodium dodecyl sulphate) sample buffer were subjected to SDS-PAGE before transfer of proteins to PVDF (polyvinylidene difluoride) membranes [62,63]. PDVF membranes were incubated overnight with the primary antibodies at 4 °C and then with specific secondary antibodies for 1 h at room temperature. Binding of the antibodies with the specific proteins has been detected by chemiluminescence.

### 4.4. Quantitative Real-Time Reverse Transcription PCR

Total RNA from C2C12 myotubes was extracted using a TRI Reagent^®®^ RNA Isolation Reagent. Then, 1–2 μg of RNA was reverse transcribed using the high capacity cDNA reverse transcription kit (Applied Biosystems, Foster City, CA, USA). TaqMan gene expression assays were used to perform real-time PCR in order to quantify the mRNA expression of S1P metabolism enzymes (SK1, SK2, SPL, SPP1, SPP2), S1PR (S1P_1_, S1P_2_, S1P_3_, S1P_4_, S1P_5_) and S1P specific transporter Spns2. Each measurement was carried out in triplicate using the CFX96 Touch™ Real-Time PCR Detection System (Biorad, Hercules, CA, USA) as described previously [64,65], by simultaneous amplification of the target sequence together with the housekeeping gene β-actin. The 2^−ΔΔCt^ method was applied as a comparative method of quantification [66], and data were normalized to β-actin expression.

### 4.5. NMR-Based Metabolomic Analyses

Conditioned media were analysed using an untargeted ^1^H-NMR-based metabolomic approach [67,68]. Samples for NMR analyses were prepared according to procedures developed to obtain highly reproducible samples for cell metabolomics [69].

Conditioned media were collected and immediately frozen at −80° C. Frozen samples were thawed in ice and shaken before use. NMR samples from conditioned media (exo-metabolome analysis) were prepared into 5.00 mm NMR tubes (Bruker BioSpin srl; Rheinstetten, Germany) by mixing an aliquot of 250 μL of a sodium phosphate buffer (70 mm Na_2_HPO_4_; 20% *v*/*v* ^2^H_2_O; 4.6 mm TMSP, pH was adjusted to the final value of 7.4 using 1 m HCl) with 250 μL of the medium.

NMR spectral acquisition and processing were performed according to optimized procedures for metabolomic analysis of growing media developed at CERM [67,68,70,71]. One-dimensional (1d) ^1^H NMR spectra were acquired on conditioned media using a Bruker 600 MHz spectrometer equipped with a 5 mm PATXI ^1^H-^13^C-^15^N and ^2^H-decoupling probe including a *z*-axis gradient coil, an automatic tuning-matching (ATM) and an automatic and refrigerate sample changer (SampleJet, Bruker BioSpin srl; Rheinstetten, Germany). ^1^H NMR spectra were acquired at 300 K with water presaturation and a 1d nuclear Overhauser enhancement spectroscopy (NOESY)- pulse sequence (noesygppr1d, Bruker). A total of 64 scans were used, with 98,304 data points, a spectral width of 18,028 Hz, an acquisition time of 2.7 s, a relaxation delay of 4 s and a mixing time of 0.1 s.

The raw data were multiplied by a 0.3 Hz exponential line broadening and Fourier transformation were applied. Transformed spectra were automatically corrected for phase and baseline distortions. The calibration of the spectra was performed to the signal of TMSP at 0.00 ppm (^1^H chemical shift).

Multivariate untargeted Principal Component Analysis (PCA) was performed on bucketed NMR spectra. To this aim, each spectrum (from 0.2 to 10.0 ppm) was segmented into 0.02 ppm chemical shift bins and the corresponding spectral areas were integrated using the AMIX software (Bruker). The area of each bin was normalized to the total spectral area, calculated with the exclusion of water region (4.5–5.0 ppm). PCA was used to obtain an overview of the dataset, i.e., visualization in a reduced space and clusters detection.

The most abundant metabolites present in the spectra were assigned and their levels analyzed. The assignment procedure was made up using a NMR spectra library of pure organic compounds, stored reference NMR spectra of metabolites and literature data. Matching between new NMR data and databases was performed using the ASSURE software (Bruker, BioSpin srl; Rheinstetten, Germany). The relative concentrations of the various metabolites were calculated by integrating the corresponding signals in the spectra using a home-made R script. The non-parametric Wilcoxon test was used for the determination of the meaningful metabolites: a *p*-value of 0.05 was considered statistically significant.

### 4.6. Electrophysiology

The membrane passive properties and transmembrane ion currents were analysed by the whole cell patch clamp technique. The patch pipettes were achieved by a vertical puller (Narishige, Tokyo, Japan) using borosilicate glass capillaries (GC150-15; Clark, Electromedical Instruments, Reading, UK). Pipettes were commonly filled with the following internal solution (mM): 130 KCl, 10 NaH_2_PO_4_, 0.2 CaCl_2_, 1 EGTA, 5 MgATP and 10 HEPES (pH 7.2 with KOH). The electrode resistance was 3–7 MΩ. Electrophysiological experiments were performed on C2C12 myotubes plated on glass coverslips and constantly superfused at a rate of 1.8 mL min^−1^ with a physiological external solution (mM): 150 NaCl, 5 KCl, 2.5 CaCl_2_, 1 MgCl_2_, 10 D-glucose and 10 HEPES (pH 7.4 with NaOH). The set-up, Axopatch 200 B amplifier, A/D-D/A interfaces Digidata 1200; Pclamp 6 software (Axon Instruments, Foster City, CA, USA) and electronic device were as described in detail in previously published papers [65,72]. The data analysis was made by Clampfit 9 (Axon Instruments, Foster City, CA, USA) software. The resting membrane potential (RMP) was evaluated in the current clamp mode of the 200 B amplifier, with I = 0. The membrane passive properties were estimated in voltage-clamp by applying a voltage pulse of ± 10 mV from a holding potential of −70 mV. The decay of the evoked passive current [34] could be fitted by the sum of 2 exponential functions. These functions represent the time course of the surface and tubular membrane passive currents, I_S_ and I_T_, respectively [73]. The related time constants are t_s_ = R_s_C_s_ and t_T_ = R_T_C_T_, where R_s_ and R_T_ represent the resistances in parallel with C_s_ and C_T_, respectively; C_s_ is the capacitance associated with the surface membrane and C_T_ is the capacitance associated with the tubular membrane. The cell linear capacitance C_m_, used as an index of the cell surface area (being the membrane-specific capacitance 1 μF/cm^2^), is calculated from the area beneath the capacitive transient current and is the overall result of C_s_ + C_T_. The membrane resistance (R_m_) values were calculated from the steady-state membrane current (I_m_) as previously reported [72].

Ion currents were evoked in voltage-clamp mode. To record the delayed rectifier K^+^ current (*I*_K_), we used the physiological external solution with nifedipine (10 µM) added to avoid the occurrence of L-type Ca^2+^ current. To record only Ca^2+^ current, *I*_Ca_, we used a Na^+^- and K^+^-free high-TEA external solution (mM): 10 CaCl_2_, 145 tetraethylammonium bromide, 10 HEPES and a suitable filling pipette solution (mM): 150 CsBr, 5 MgCl_2_, 10 Ethylene-bis(oxyethylenenitrilo) tetraacetic acid (EGTA), 10 (4-(2-hydroxyethyl)-1-piperazineethanesulfonic acid) (HEPES) (pH = 7.2) [74]. In any case, we applied a pulse protocol of stimulation consisting of 1-s step voltage pulses, ranging from −80 to 50 mV, in 10 mV increments starting from a HP = −60 mV for *I*_K_ or −80 mV for *I*_Ca_. P4 procedure was used to remove on-line capacitive and leak currents. The current amplitude values were normalized to C_m_ to have a correct evaluation between records acquired from cells of different size. Thus, the ratio I/C_m_ (in pA/pF) is intended as current density. All drugs were from Sigma Chemical (St. Louis, MO, USA). The experiments were performed at room temperature (22 °C).

### 4.7. Statistical Analysis

To perform densitometric analysis of the Western blot bands and graphical representations, ImageJ software and GraphPad Prism 6.0 (GraphPad Software, San Diego, CA, USA) were utilized, respectively. Statistical analysis was performed using Student’s t test, one-way and two way ANOVA followed by Bonferroni’s post hoc test and paired Wilcoxon test.

## Data Availability

Data is contained within the article or supplementary material.

## Supplementary Materials

Supplementary Materials can be found at https://www.mdpi.com/1422-0067/22/3/1280/s1.

## Abbreviations

S1P	sphingosine 1-phosphate
TNFα	tumor necrosis factor alpha
S1PR	sphingosine 1-phosphate receptors
SK	sphingosine kinase
SPL	S1P lyase
SPP	S1P phosphatases
LPP	lipid phosphate phosphatases
Spns2	spinster homolog 2
NMR	Nuclear magnetic resonance
RMP	resting membrane potential
R_m_	membrane resistance
C_m_	cell capacitance
PCA	principal component analysis
