# Supplementary material for: Role of Sphingosine 1-Phosphate Signalling Axis in Muscle Atrophy Induced by TNFα in C2C12 Myotubes
Source: Int J Mol Sci. 2021 Jan 28;22(3):1280. doi: 10.3390/ijms22031280 (PMC7866171; doi:10.3390/ijms22031280)
Supplement: Supplementary file 1 [file ijms-22-01280-s001.pdf]

Supplementary Materials

Table S1. Effect of TNFα on myotubes passive properties

	Control	TNFα	PF-543	PF-543+TNFα	VPC23019	VPC23019+TNFα
**RMP (mV)**	-41.0±3.7	-27.8±0.98***	-28.2±3.6*	-25.6±8.9*	-28.8±9.6*	-23.5±2.8*
	(n=8)	(n=6)	(n=6)	(n=6)	(n=13)	(n=8)
**C_m (pF)**	26.4±19.6	3.5±2.1*	41.5±24.6§	57.6±20.1§	62.8 ±51§	53.1±25§
	(n=8)	(n=18)	(n=12)	(n=9)	(n=10)	(n=6)
**R_m (MΩ)**	524.1±157.5	309.8±34.3*	584.5±197.2§	498.4±177.9*§	535.8±400	851.7±394
	(n=17)	(n=10)	(n=16)	(n=14)	(n=6)	(n=6)

	Control	TNFα	PF-543	PF-543+TNFα	VPC23019	VPC23019+TNFα
C_T/C_s	4.5±1.2	2.1±0.9*	4.5±1.8§	5.5±2.3§	3.5 ±2.7	4.8 ±1.05§
	(n=10)	(n=18)	(n=9)	(n=9)	(n=14)	(n=5)

Table S1. All of the results are as mean ± SD. 'n' is the number of cells included in the statistical analysis. *Indicates $p<0.05$ vs control; *** indicates $p<0.001$ vs control (Student's T test). § indicates $p<0.05$ vs TNFα (One-way ANOVA and Bonferroni's *post hoc* test).

Figure S1

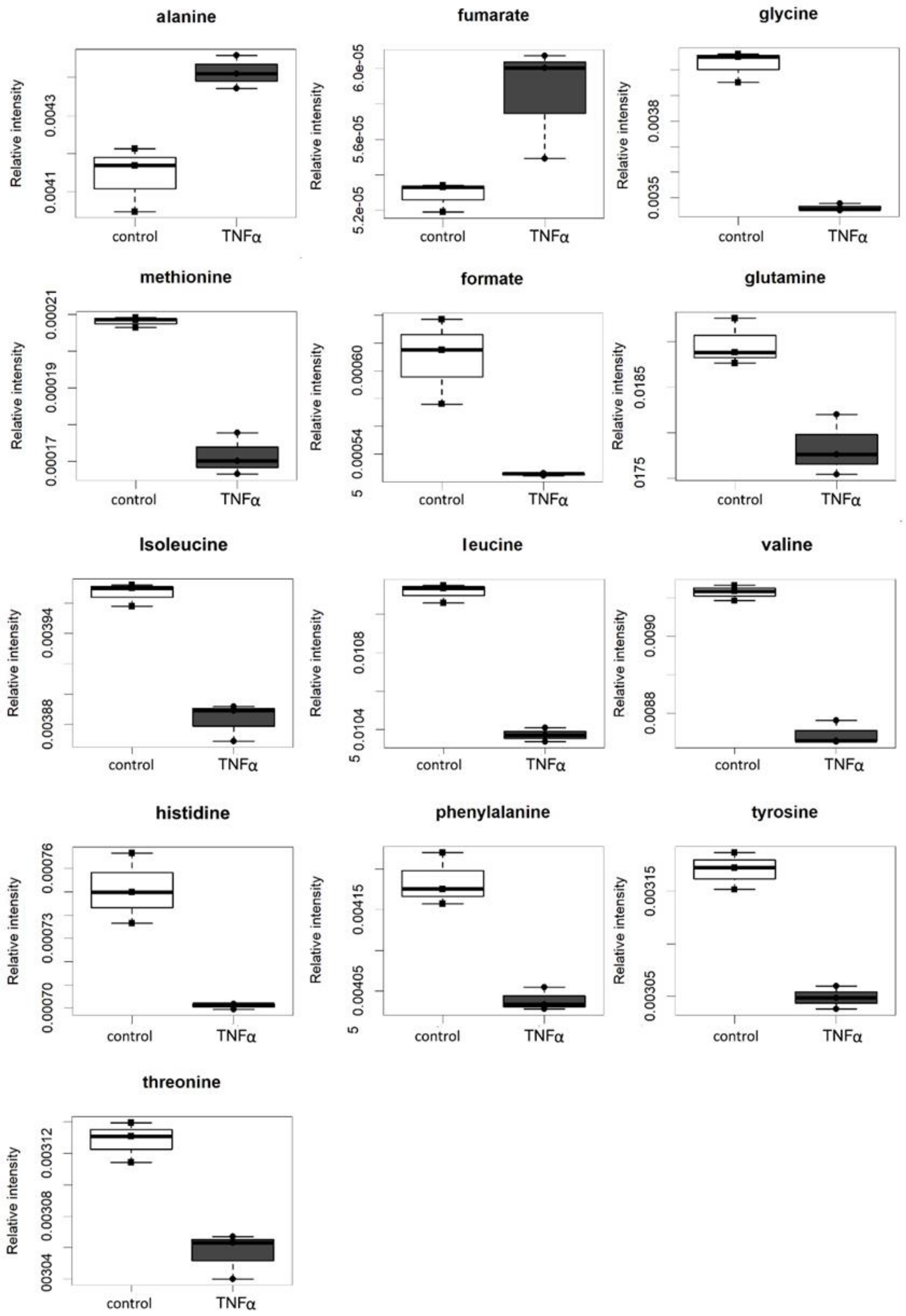

Figure S1. Boxplot representing metabolites levels in the different group of samples, i.e. white: Control; dark gray: TNFα.

Figure S2

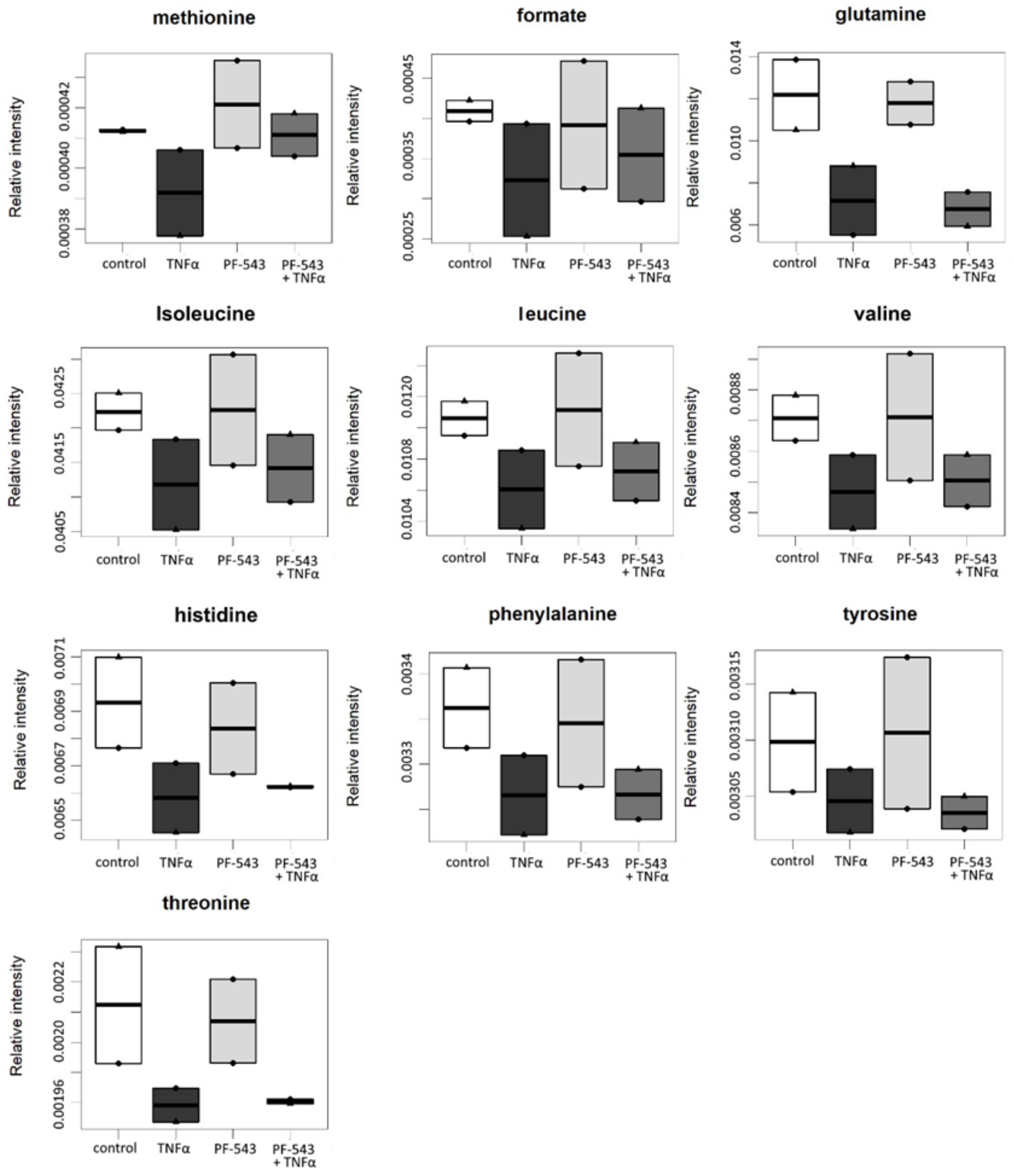

Figure S2. Boxplot representing metabolites levels in the different group of samples, i.e. white: Control; dark gray: TNFα; light gray: PF-543; medium gray: P5-543+TNFα.

Figure S3

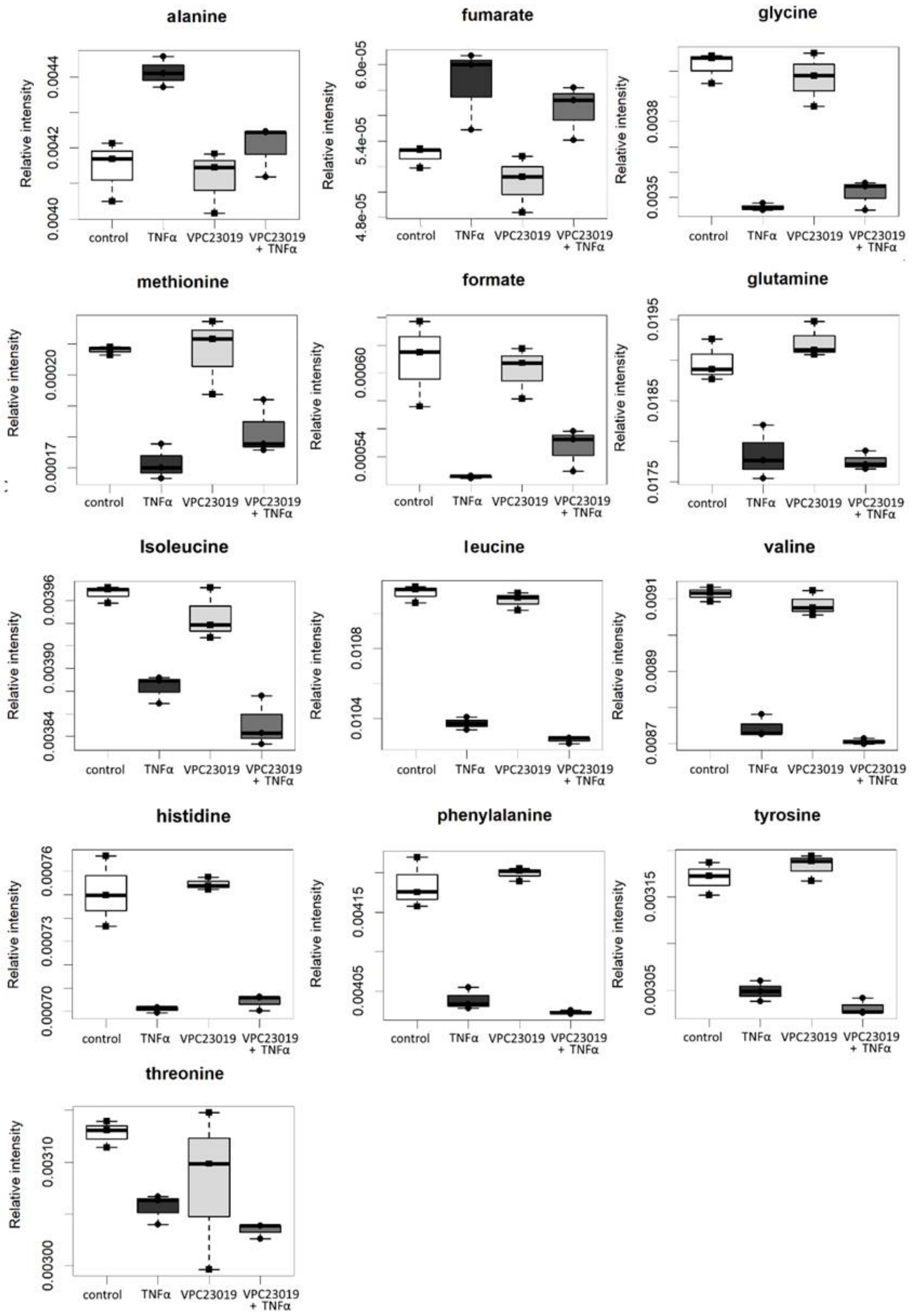

Figure S3. Boxplot representing metabolites levels in the different group of samples, i.e. white: Control; dark gray: TNFα; light gray: VPC23019; medium gray: VPC23019+TNFα.
